# Cytoarchitecture, intersubject variability, and 3D mapping of four new areas of the human anterior prefrontal cortex

**DOI:** 10.3389/fnana.2022.915877

**Published:** 2022-08-11

**Authors:** Ariane Bruno, Sebastian Bludau, Hartmut Mohlberg, Katrin Amunts

**Affiliations:** ^1^Institute of Neuroscience and Medicine (INM-1), Research Centre Jülich, Jülich, Germany; ^2^Cécile and Oskar Vogt Institute for Brain Research, University Hospital Düsseldorf, Medical Faculty, Heinrich-Heine-University Düsseldorf, Düsseldorf, Germany

**Keywords:** dorsolateral prefrontal cortex (DLPFC), brain mapping, human brain atlas, Julich-brain, cerebral cortex

## Abstract

The dorsolateral prefrontal cortex (DLPFC) plays a key role in cognitive control and executive functions, including working memory, attention, value encoding, decision making, monitoring, and controlling behavioral strategies. However, the relationships between this variety of functions and the underlying cortical areas, which specifically contribute to these functions, are not yet well-understood. Existing microstructural maps differ in the number, localization, and extent of areas of the DLPFC. Moreover, there is a considerable intersubject variability both in the sulcal pattern and in the microstructure of this region, which impedes comparison with functional neuroimaging studies. The aim of this study was to provide microstructural, cytoarchitectonic maps of the human anterior DLPFC in 3D space. Therefore, we analyzed 10 human post-mortem brains and mapped their borders using a well-established approach based on statistical image analysis. Four new areas (i.e., SFS1, SFS2, MFG1, and MFG2) were identified in serial, cell-body stained brain sections that occupy the anterior superior frontal sulcus and middle frontal gyrus, i.e., a region corresponding to parts of Brodmann areas 9 and 46. Differences between areas in cytoarchitecture were captured using gray level index profiles, reflecting changes in the volume fraction of cell bodies from the surface of the brain to the cortex-white matter border. A hierarchical cluster analysis of these profiles indicated that areas of the anterior DLPFC displayed higher cytoarchitectonic similarity between each other than to areas of the neighboring frontal pole (areas Fp1 and Fp2), Broca's region (areas 44 and 45) of the ventral prefrontal cortex, and posterior DLPFC areas (8d1, 8d2, 8v1, and 8v2). Area-specific, cytoarchitectonic differences were found between the brains of males and females. The individual areas were 3D-reconstructed, and probability maps were created in the MNI Colin27 and ICBM152casym reference spaces to take the variability of areas in stereotaxic space into account. The new maps contribute to Julich-Brain and are publicly available as a resource for studying neuroimaging data, helping to clarify the functional and organizational principles of the human prefrontal cortex.

## Introduction

**Figure 1:**
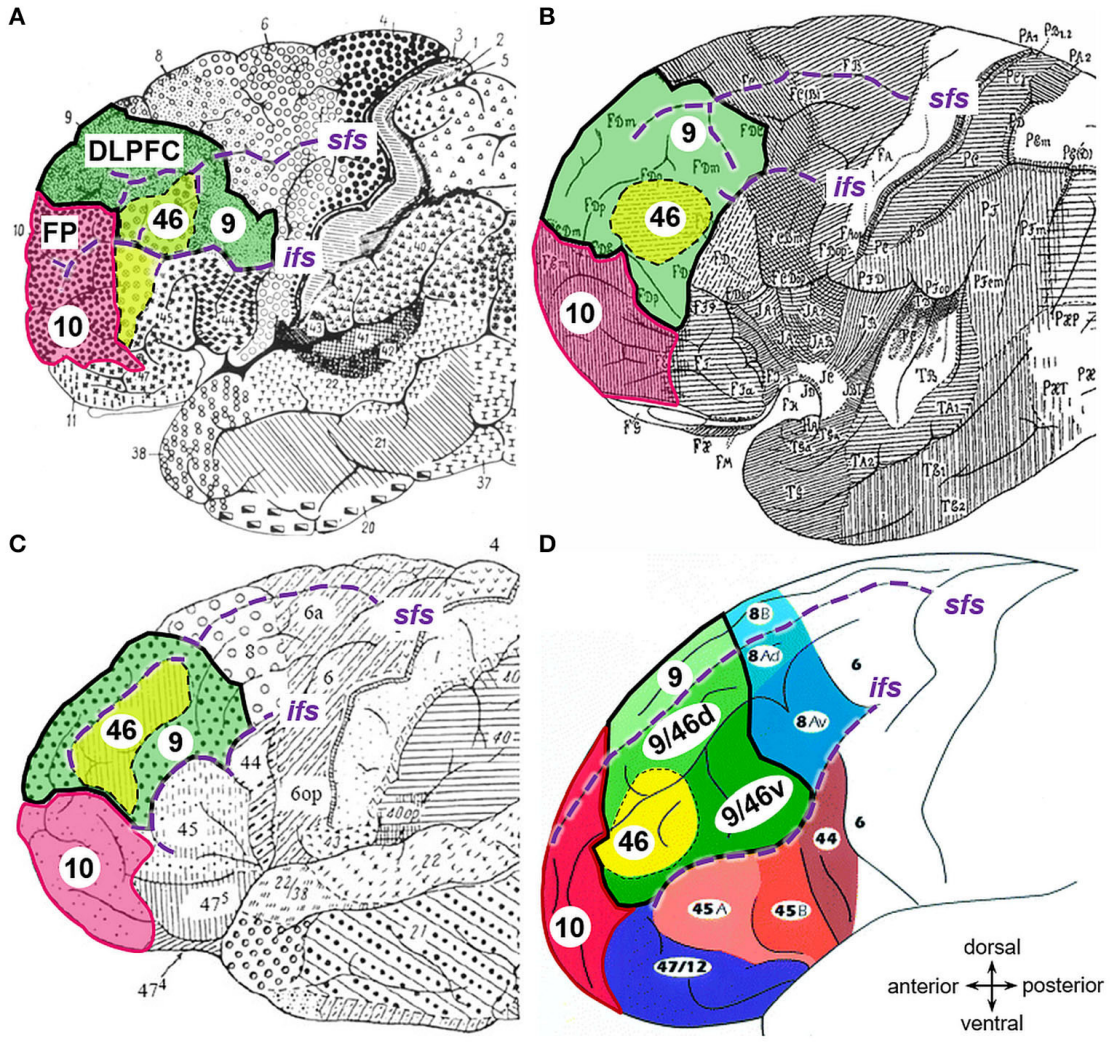
Maps of the prefrontal cortex. Adapted cytoarchitectonic maps showing the dorsolateral prefrontal cortex (DLPFC, bold black lines) and frontal pole (Fp, magenta) as segmented and labeled by **(A)** Brodmann (1909), **(B)** von Economo and Koskinas (1925), **(C)** Sarkissov et al. (1955), and **(D)** Petrides and Pandya (1999) with copyright permission. Related areas are highlighted in corresponding colors: area 9 (green) and 46 (yellow) of the DLPFC and area 10 of the Fp (magenta). Sulci labeled in violet italic (*sfs*, superior frontal sulcus; *ifs*, inferior frontal sulcus).

The human prefrontal cortex is thought to be crucial for processing executive functions and has, thus, become a major target for clinical and neuropsychological studies (Jones and Graff-Radford, 2021; Friedman and Robbins, 2022). The cortex exhibits a high degree of folding and has undergone considerable changes during evolution (Wise, 2008; Preuss and Wise, 2022). It can be subdivided into an orbitofrontal, medial, ventrolateral, and dorsolateral prefrontal region (DLPFC; see Figure 1; Fuster, 2001). The DLPFC plays a key role in specific mechanisms of cognitive control and behavior (Wise, 2008; Friedman and Robbins, 2022), which includes executive functions, such as monitoring (O'Reilly, 2010), controlling behavioral strategies (Shallice and Burgess, 1991; Barraclough et al., 2004; Sallet et al., 2013), planning of actions (Cieslik et al., 2013), attentional selection (Rowe and Passingham, 2001; Hoshi and Tanji, 2004; Vossel et al., 2014), value encoding (Kouneiher et al., 2009; Sokol-Hessner et al., 2012), decision making (Philiastides et al., 2011; Rahnev et al., 2016), and working memory (Petrides, 2000; Rowe et al., 2000). Functionally, the DLPFC seems to be divided along an anterior-posterior and dorsal-ventral axis (O'Reilly, 2010; Goulas et al., 2012; Sallet et al., 2013; Badre and Nee, 2018). The anterior part of the DLPFC is activated with increasingly abstract representations and complex actions as needed for action inhibition processes and conflict resolution (Cieslik et al., 2013). In contrast, the posterior region was more associated with working memory and action execution (Cieslik et al., 2013).

Disorders like schizophrenia (Smucny et al., 2022), obsessive-compulsive disorder (Ahmari and Rauch, 2022), depression (Stockmeier and Rajkowska, 2004; Zuo et al., 2018), and bipolar disorder (Zhang et al., 2020) are associated with deficits in executive functions, which are linked to alterations in the DLPFC and associated circuitry (Menon, 2011; Snyder et al., 2015; Wilczynska et al., 2018). Several studies suggest that, inter alia, cellular changes in DLPFC are responsible for the behavioral and functional impairments related to schizophrenia (Smucny et al., 2022) and, accordingly, abnormalities in pyramidal neurons in the deeper layer III of the DLPFC were found (Pierri et al., 2001). These show smaller soma sizes, reduced axonal arbors, and shorter basilar dendrites (Volk and Lewis, 2010). Such morphologic cellular changes of the DLPFC were also detected in depression and bipolar disorder, where the density of glia and neurons was reduced in a lamina-specific way (Rajkowska, 2000).

In addition to alterations at the cellular scale, several neuropsychiatric diseases correlate with DLPFC changes at the molecular and genetic levels. For example, the expression of some microglial genes was downregulated in patients suffering from bipolar disorder (Zhang et al., 2020). Since the symptomatology and prevalence differ between males and females in several of these diseases, it has been hypothesized that sex differences are also present in structural and molecular characteristics (Gur and Gur, 2017). Indeed, such differences were identified by both single studies and meta-analyses, ranging from differences in gene expression (Zhang et al., 2020) to structural alterations, like gray matter volume and cortical thickness (Ruigrok et al., 2014; Ritchie et al., 2018). Regarding the DLPFC, sex differences have been examined mainly *via* neuroimaging approaches (e.g., gray matter volume; Lotze et al., 2019). However, little is known at a high-resolution microstructural level.

Due to the key role of the DLPFC in higher cognitive functions and its involvement in a plethora of neurological disorders, many attempts to create histological maps of this structure have been performed in the past. At the beginning of the twentieth century, Brodmann (1909) created one of the first microanatomical parcellations to unravel the structural-functional correlations of the human brain. He segregated the human cortex into more than 40 areas, and divided the DLPFC into two distinct cytoarchitectonic areas, namely BA9 and BA46 (Brodmann, 1909). According to this map, BA9 occupies the whole superior frontal gyrus, the dorsorostral part of DLPFC, and the caudal parts of the middle frontal gyrus (*mfg*). BA46 is located on the remaining portion of the *mfg* and the inferior frontal gyrus bordering ventrally to BA45 (Figure 1A). However, the two-dimensional schematic map of Brodmann only illustrates the superficially exposed areas of one “typical” brain. Thereby, intersubject variability is not addressed, and area localization and extent are not defined within the sulci, although two-thirds of the cerebral cortex is hidden in these grooves (Zilles et al., 1997). Additionally, classifications based on the cytoarchitecture differ considerably between parcellation attempts of different researchers (Brodmann, 1909; von Economo and Koskinas, 1925; Sarkissov et al., 1955; Rajkowska and Goldman-Rakic, 1995a,b; Petrides and Pandya, 1999). The maps of von Economo and Koskinas (1925), as well as from Sarkissov et al. (1955), placed area 46 exclusively within the *mfg*, surrounded by area 9 (labeled as FD in the map of von Economo and Koskinas) like an “island,” and, therefore, not bordered by area 45 (Figures 1B,C). Rajkowska and Goldman-Rakic (1995a) firstly defined the so-called transitional areas as cortical regions showing both cytoarchitectonic features from adjacent areas. For instance, transitional area 9–46 lies in the depths of the superior and middle frontal sulci, thus, in-between area 9 and area 46 (Rajkowska and Goldman-Rakic, 1995b). Petrides and Pandya (1999) published a similar cytoarchitectonic map, including transition areas (Figure 1D). In addition to the cytoarchitectonic characterization, Rajkowska and Goldman-Rakic (1995b) reconstructed and superimposed their areas 9 and 46 into the Talairach and Tournoux's (1997) coordinate space for five cases.

Thus, previous cytoarchitectonic maps show considerable discrepancies in the location and distribution of DLPFC sub-areas and their relationship to sulci and gyri. Reasons may include differing and partly subjective criteria used to define and delineate subregions and the analysis of rather small samples in such time- and labor-intensive studies on microstructure, while differences in the sulcal pattern of the DLPFC are considerable (Ono et al., 1990; Miller et al., 2021a). The DLPFC exhibits a high variability of sulci, i.e., the middle frontal sulcus (*mfs*), which divides the *mfg* into a dorsal and ventral part, existing only in 86% of human brains (Ono et al., 1990). Furthermore, individuals possessing an *mfs* to show remarkable variation in its structure, either as a large main furrow parallel to the superior and inferior frontal sulcus or as several irregular smaller (so-called tertiary) sulci (Vogt and Vogt, 1926; Huttner, 2004; Miller et al., 2021a).

In addition to these macroscopic challenges, most maps do not allow a direct superimposition with three-dimensional (3D) datasets of functional imaging studies, a prerequisite for their direct comparison (Zilles and Amunts, 2010). Parcellations of the cortex obtained from MRI-based studies suggest rather detailed segregation of the prefrontal cortex (Goulas et al., 2012; Cieslik et al., 2013; Sallet et al., 2013; Glasser et al., 2016b; Donahue et al., 2018), for which microstructural, cytoarchitectonic correlates, however, have not yet been identified.

Consequently, a more detailed anatomical reference of the human DLPFC is mandatory that accounts for intersubject variability. Hence, this study aimed to delineate and cytoarchitectonically analyze the human DLPFC, focusing on the anterior superior frontal sulcus (*sfs*) and *mfg* to provide cytoarchitectonic correlates for the diverse functions commonly linked with this brain region. We applied a quantitative, architectonic approach analyzing the laminar cell-body distribution (Schleicher et al., 2009; Amunts et al., 2020) to detect local changes in cytoarchitecture for the definition of borders in serial histological sections of post-mortem brains. This method has been applied to numerous cortical areas, including motor and sensory areas, as well as association areas (Amunts et al., 2020). The comparability of functional imaging studies with the microstructural data and border delineation in the same reference space is a critical aspect of this study. As interindividual variability influencing the cytoarchitectonic parcellations is a challenge for that aspect, probability maps were created for each area based on the analysis of ten brains. These probability brain maps assess the variance between individual brains. Finally, we calculated area volumes and volume fractions of cell bodies in the newly identified areas and checked these measures for potential interhemispheric and sex differences because of the involvement of this brain region in neuropsychiatric disorders.

## Materials and methods

### Histological processing of post-mortem brains

Ten brains (five men, five women, age range 30–86 years, mean 66 (men: 56.6 years; women: 76.2 years) were obtained from the Body Donor Program of the Department of Anatomy of the University of Düsseldorf (Table 1). Ethics approval and written informed consent were obtained (medical faculty, Heinrich-Heine-University Düsseldorf, Germany, ethics approval number 4863). Clinical records did not show any history of psychiatric or neurological diseases.

**Table 1:** Post-mortem brains obtained from the Body Donor Program were used for the cytoarchitectonic analysis of the anterior DLPFC.

**Brain no**.	**Sex**	**Age [years]**	**Cause of death**	**Fresh brain weight [g]**
BC04	Male	75	Acute glomerulonephritis	1,349
BC05	Female	59	Cardiorespiratory insufficiency	1,142
BC08	Female	72	Renal failure	1,216
BC09	Female	79	Cardiorespiratory insufficiency	1,110
BC10	Female	85	Mesenteric infarction	1,046
BC11	Male	74	Myocardial infarction	1,381
BC13	Male	39	Drowning	1,234
BC14	Female	86	Cardiorespiratory insufficiency	1,113
BC20	Male	65	Cardiorespiratory insufficiency	1,392
BC21	Male	30	Bronchopneumonia	1,409

The histological procedure, 3D reconstruction, and subsequent image analysis were performed as previously described in detail (Amunts et al., 2020). Briefly, brains were extracted <24-h post-mortem and fixed for at least 3 months in formalin or Bodian's fixative. Magnetic resonance imaging (MRI) was conducted to record the original shape and size of the brains using a T1-weighted 3D FLASH sequence with a Siemens 1.5 Tesla scanner (Erlangen, Germany). Images were used to correct later for distortions and create 3D reconstructions of the histological sections as described previously. The whole paraffin-embedded brains were cut in serial, coronal sections with a thickness of 20 μm. Each 15th section was stained for cell bodies using a modified silver staining method (Merker, 1983) and was digitized on a flatbed scanner. At least every 60th section was analyzed, resulting in a maximal distance of 1.2 mm between them.

### Observer-independent detection of cytoarchitectonic borders using the gray level index (GLI)

**Figure 2:**
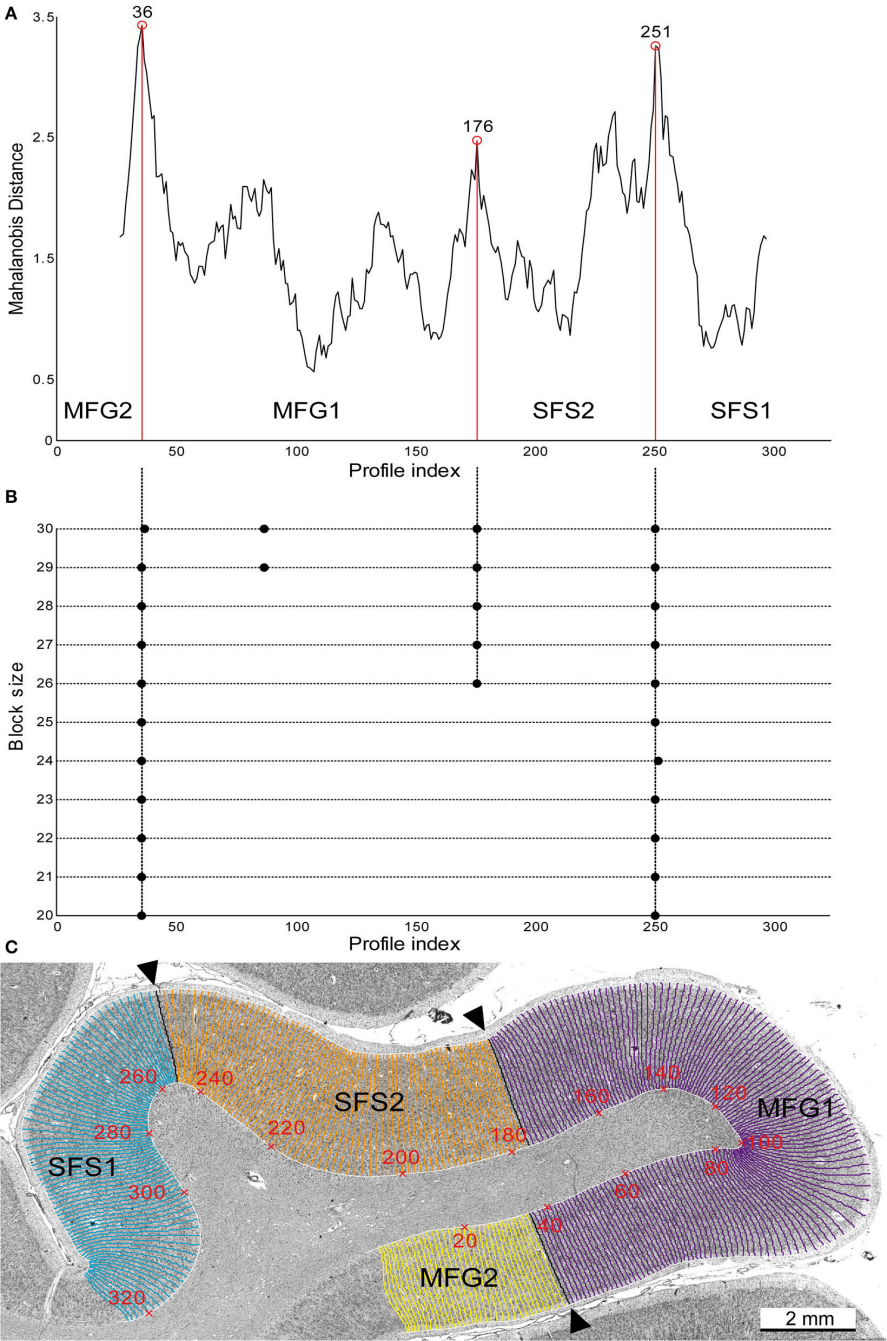
Observer-independent border detection. Significant maxima of the Mahalanobis distance (MD) at profile numbers 36, 176, and 251 (labeled with red circles) are plotted against the profile index **(A)**. These positions indicate the borders between SFS2 and MFG1 and the neighboring areas SFS1 and MFG2 **(A,C)**. Significant maxima (indicated by black dots) were tested for different block sizes (*n* = 20–30) and accepted as significant borders when they were found for at least three block sizes **(B)**. Corresponding histological image of brain BC09 depicting the newly identified areas **(C)**. GLI profiles were calculated along traverses (numbered in red), representing cytoarchitecture changes from the border of layers I/II to the layer VI/white matter border. The observer-independent defined borders, corresponding to maxima of the MD function shown in **(A)**, are marked with black arrowheads.

**Figure 3:**
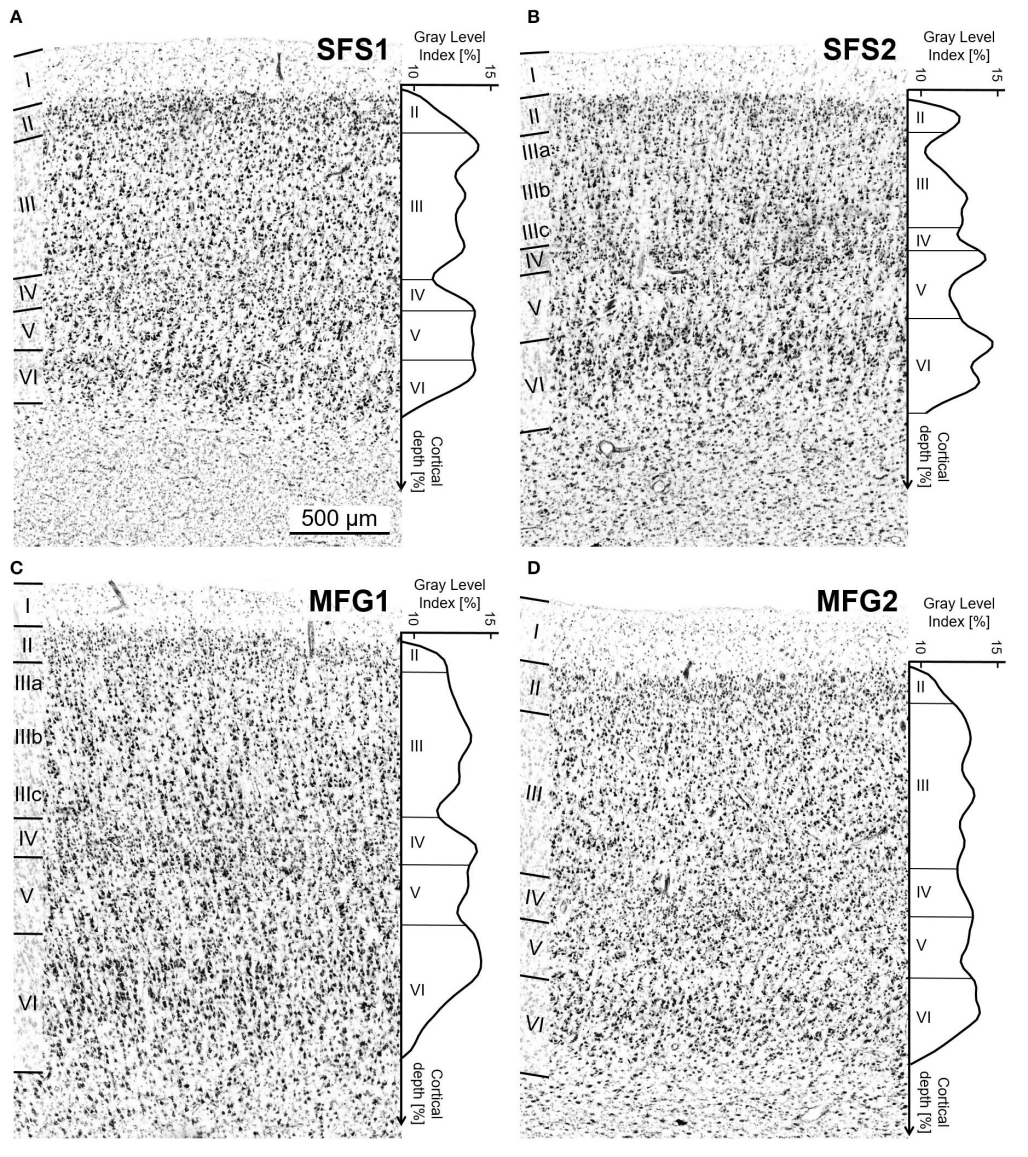
Cytoarchitecture of areas SFS1, SFS2, MFG1, and MFG2 with corresponding mean GLI profiles. The GLI profiles, next to the histologic images, reflect the laminar changes in the volume fraction of cell bodies and, thus, the distinct cytoarchitecture. The statistical image analysis was based on these GLI profiles. Area SFS1 was characterized by a cell dense layer III with medium-sized pyramidal cells and a well-developed layer IV compared to neighboring areas BA9 and SFS2. Layer V was not subdividable, and the border between the thin layer VI and the white matter was sharp **(A)**. The most characteristic criteria for identifying area SFS2 was the thin blurry layer IV due to large pyramidal cells in deeper layer III and upper layer V. Layer IIIa was loosely packed, and layer VI was very prominent with large cells **(B)**. Typical for area MFG1 were large pyramidal cells in deeper layer III and upper layer V. Layer VI was prominent and cell-rich with a blurry transition to the white matter **(C)**. Area MFG2 was characterized by a relative homogenous cell size across all layers, a broad well-developed layer IV, and a sharp transition to white matter **(D)**. Contrast of histological images was enhanced for better visualization. Scale bar 500 μm in **(A)** refers to all **(A–D)**.

Considering the large size of the DLPFC, we investigated the cortex with a focus on the anterior *sfs* and rostral aspects of the *mfg*. An observer-independent approach was employed to identify borders between microscopically distinct areas (Figure 2; Schleicher et al., 1999, 2005, 2009). The cytoarchitecture and cortical borders were analyzed in rectangular regions of interest (ROI). ROIs were digitized with a CCD camera (Axiocam MRm, ZEISS, Germany) connected to an optical light microscope (AxioObserver.Z1, ZEISS, Germany). The camera and the computer-controlled motorized stage of the microscope (Axioplan 2 imaging, ZEISS, Germany), with an in-plane resolution of 1.02 μm per pixel, were operated by the Zeiss image analysis software Axiovision (version.4.6). We used a Matlab-based script (The MathWorks, Inc., Natick, MA, USA) to convert the digitized ROIs into gray level index (GLI) images (Schleicher et al., 2009). The GLI estimates the volume fraction of cell bodies (Wree et al., 1982) in a measuring field of 16 × 16 pixels and, thus, represents the cytoarchitectonic organization (Schleicher et al., 1986; Bludau et al., 2014). The outer (between layers I and II) and inner (between layer VI and the white matter) lines were defined interactively in each GLI image using in-house software written in MatLab. A physical model based on electric field lines (Jones et al., 2000) was used to calculate curvilinear traverses running perpendicular to the cortical layers (Schleicher et al., 2009). GLI values from the surface to the white matter were extracted along those traverses and led to GLI profiles (Figure 2C). The shape of these GLI profiles describes changes in GLI values from the cortical surface to the white matter and, thus, mirrors laminar changes in cytoarchitecture (Schleicher et al., 2009; Figure 3). Because cortical thickness varied between brains and brain regions, each GLI profile was adjusted to a cortical depth of 100%. The shape of the GLI profiles was parameterized by 10 features (i.e., mean GLI value, standard deviation, skewness, cortical depth of the center of gravity, kurtosis, and analogous parameters of the profile's first derivatives). Features were combined into a feature vector (Schleicher et al., 2009). For the observer-independent border detection, the shapes of adjacent profiles, reflected by this ten-dimensional feature vector, were compared. Twelve to 30 profiles were pooled into a profile block. Differences between feature vectors of neighboring blocks of GLI profiles reflecting laminar differences in cytoarchitecture were calculated using the Mahalanobis distance (MD) (Mahalanobis et al., 1949; Schleicher et al., 2000) with the subsequent Hotelling's *T*^2^ test (Bonferroni corrected alpha level of 0.001; Figure 2A). Distances were calculated using a sliding window approach, where blocks of profiles were stepwise moved along the cortical ribbon, whereby the procedure was executed for all block sizes between 12 and 30 profiles per block (Schleicher and Zilles, 1990; Schleicher et al., 2005). Areal borders were accepted when significant local maxima of the distance function were detected at the same position across several block sizes and reproducible in at least three adjacent histological sections (Figure 2B).

### Reconstruction of cortical areas and stereotaxic maps

The borders of the identified new areas were labeled over their full extent in digitized high-resolution scans of serial histological sections *via* the in-house developed “Section tracer online tool.” Subsequently, cytoarchitectonic areas were 3D-reconstructed using the same deformation fields as calculated for the histological volumes of the post-mortem brains (Amunts et al., 2020). The 3D-reconstructed maps of each brain were then spatially normalized to the reference space of the single-subject template of the Montreal Neurological Institute (“Colin27”) and the non-linear asymmetric MNI152 2009c template (ICBM152casym; Evans et al., 2012) using a combination of linear and non-linear elastic registration (Amunts et al., 2020). Then, the areas of all 10 brains were superimposed in the two MNI reference spaces, which resulted in a probability map of each area in stereotaxic space (Amunts et al., 2020). The probability maps indicate the intersubject variability of the cortical area at a particular position in the reference brain. Probabilities were color-coded, ranging from dark blue (low probability) to red (high probability).

Based on these probability maps, a continuous, non-overlapping maximum probability map (MPM) of the newly identified and prior mapped areas was generated, in which each voxel was assigned to the area with the highest probability for this particular voxel (Eickhoff et al., 2005). Subsequently, the centers of gravity were computed in each of the two spaces.

The areal representations were included in the Julich-Brain Atlas (https://julich-brain-atlas.de/), as well as in the HBP atlas as part of the EBRAINS research infrastructure (https://ebrains.eu/service/human-brain-atlas/), and are publicly available.

### Volumetric analysis of delineated areas

Volumes of the new areas were analyzed and compared between brains regarding interhemispheric and sex differences. An individual correction factor to account for tissue shrinkage during histological processing was calculated for each post-mortem brain based on the ratio between fresh brain volume and brain volume after histological treatment (Amunts et al., 2007). As brains differ in size, area volumes were normalized to individual whole-brain volumes to compare volumes of DLPFC areas (Bludau et al., 2014). We analyzed normalized volumes with a mixed model ANOVA with a repeated-measures design (within factors, area and hemisphere; between factor, sex) was used to test for significance in volume. Normality was checked by a Shapiro–Wilk test and Sphericity by the Mauchly's test. The error variances were homogeneous, as assessed by the Levene's test. A significance level of α = 0.05 was set for all tests.

### GLI as an indicator of the volume fraction of cell bodies

The GLI was analyzed to examine putative sex differences. An increased volume fraction of cell bodies is equivalent to a decreased proportion of neuropil, i.e., a smaller proportion of space covered by axons, dendrites, and synapses. Mean GLI values were computed based on 15–20 profiles in three histological sections per area, hemisphere, and brain. These mean GLI values were used to analyze sex, interhemispheric, and inter-area differences for all identified new areas. Statistical analyses with a significance level of α = 0.05 were performed with a mixed-model ANOVA with a repeated-measures design (within factors, area and hemisphere; between factor, sex) as described in the volumetric analysis section.

### Hierarchical clustering of mean areal GLI profiles

A hierarchical cluster analysis was performed to detect dissimilarities between the new anterior DLPFC areas and neighboring frontal pole areas of Fp1 and Fp2 (Bludau et al., 2014), areas 44 and 45 of the Broca's region (Amunts et al., 2004), and posterior DLPFC areas (Amunts et al., 2021) described before. Mean GLI profiles based on 15–20 individual profiles taken from three sections, where areas showed a nearly horizontally layered cortex, were calculated for each area in both hemispheres. Based on these mean GLI profiles, feature vectors for each area were generated, and discriminant analyses were calculated using the Euclidian distance and the Ward-linking method (Ward, 1963). A high Euclidian distance indicates a large degree of cytoarchitectonic difference and a low structural similarity and vice versa.

## Results

**Figure 4:**
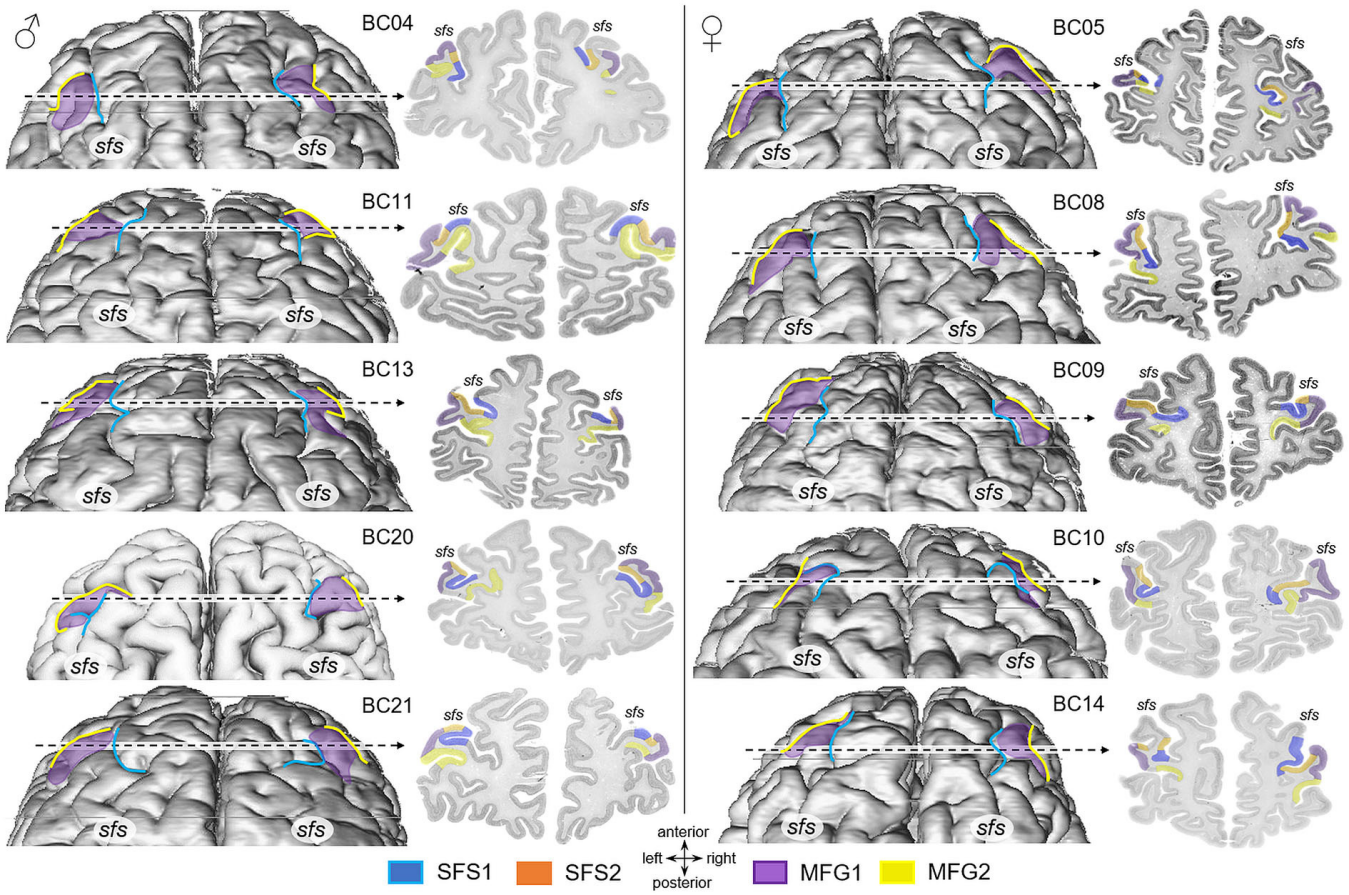
Dorsal views of 3D area reconstructions of ten individual brains. Dorsal surface reconstruction of areas SFS1 (blue), MFG1 (purple), and MFG2 (yellow) separated by sex (male brains: left, female brains: right) showing the interindividual variability concerning differences in size and shape of areas and variability in the sulcal pattern. Area SFS2 was excluded from the reconstruction for visualization reasons. The dotted line indicates the localization and extent of cytoarchitectonic areas SFS1, SFS2, MFG1, and MFG2 on the corresponding histological section. Area SFS1 was predominately situated in the superior frontal sulcus (*sfs*) depth. Area SFS2 joined SFS1 in the sulcus and reached the surface of the anterior part of the middle frontal gyrus, occupied by the area MFG1. Adjacent to area MFG1, area MFG2 reached into the ventrally neighboring sulcus.

Four new cytoarchitectonic areas were identified within the anterior DLPFC (Figure 3). According to their location in the *sfs* and on the *mfg*, the areas were labeled SFS1 (superior frontal sulcus 1), SFS2 (superior frontal sulcus 2), MFG1 (middle frontal gyrus 1), and MFG2 (middle frontal gyrus 2). Figure 4 depicts the considerable intersubject variability in the sulcal pattern, localization, and extent of the new areas in the dorsal surface reconstruction of 10 individual brains. Examinations of the 3D area reconstructions revealed that the cytoarchitectonically delineated boundaries between areas do not consistently correspond to the sulcal contours. Area SFS1 was primarily located within the depth of the *sfs* but also partly extended to the descending and ascending bank of the *sfs*. Area SFS2 was located ventrally to area SFS1, on the ascending ventral bank of the *sfs* and partly reaching the surface of the *mfg*. Ventral to area SFS2, MFG1 covered mainly the surface of the anterior *mfg*. Adjacent to area MFG1, area MFG2 reached into the ventrally neighboring sulcus, which was either the extension of the fronto-marginal sulcus (for example, see BC05 Figure 4) or, if existing, the anterior beginning of the *mfs* (for example, see BC04 Figure 4). An *mfs* was present in 17 of 20 examined hemispheres (Figure 4).

### Cytoarchitecture

The anterior DLPFC areas SFS1, SFS2, MFG1, and MFG2 were adjacent to putative BA9, Fp1, and BA46. Cytoarchitectonic criteria for Fp1, BA9, and BA46 were taken from the publications by Bludau et al. (2014) and Rajkowska and Goldman-Rakic (1995a), respectively. The characteristic cytoarchitecture of all areas is summarized in Table 2.

**Table 2:** Cytoarchitectonic characteristics of anterior DLPFC areas SFS1, SFS2, MFG1, and MFG2 and neighboring areas.

**Area**	**Cytoarchitectonic characteristics**
SFS1	Homogenous appearance
	Uniformly packed layer III
	Dense, well-definable layer IV compared to neighboring areas BA9 and SFS2
	Sharp border between layer VI and white matter compared to BA9
SFS2	Sparser cell packing than in SFS1
	Large pyramidal cells in deeper (IIIc) than in upper (IIIa) layer III compared to SFS1
	Blurry, not well-definable layer IV compared to areas SFS1, MFG1, and MFG2
MFG1	Large pyramidal cells in deeper layer IIIc than in SFS2
	Broader layer IV than in SFS2 but not as dense as compared to MFG2
	Prominent layer VI with large cells and blurry border to white matter
MFG2	Uniform appearance due to homogenous cell density and cell size
	Dense, prominent layer II than in MFG1 but not as in SFS1
	Broad, well-developed, dense layer IV
	Sharp border between layer VI and white matter
Fp1	Sharp border between layers I, II, and III
	Dense layer II and deeper layer III
	Considerably larger pyramidal cells in deeper than in upper layer III
	Layer IV is not as broad as in SFS1
BA9	Medium-sized (IIIa and IIIb) and large (IIIc) pyramidal cells in layer III
	Narrower layer IV than in SFS1
	Layer V can be separated in Va with large pyramidal cells and in a pale Vb
	Indistinct border between layer VI and white matter
BA46	Thin layer II
	Slight cell size gradient across layer III
	Densely packed layer IV
	Layer V with its medium-sized pyramidal cells is more prominent as in MFG2
	Broader layer VI and blurry white matter border compared to MFG2

**Figure 5:**
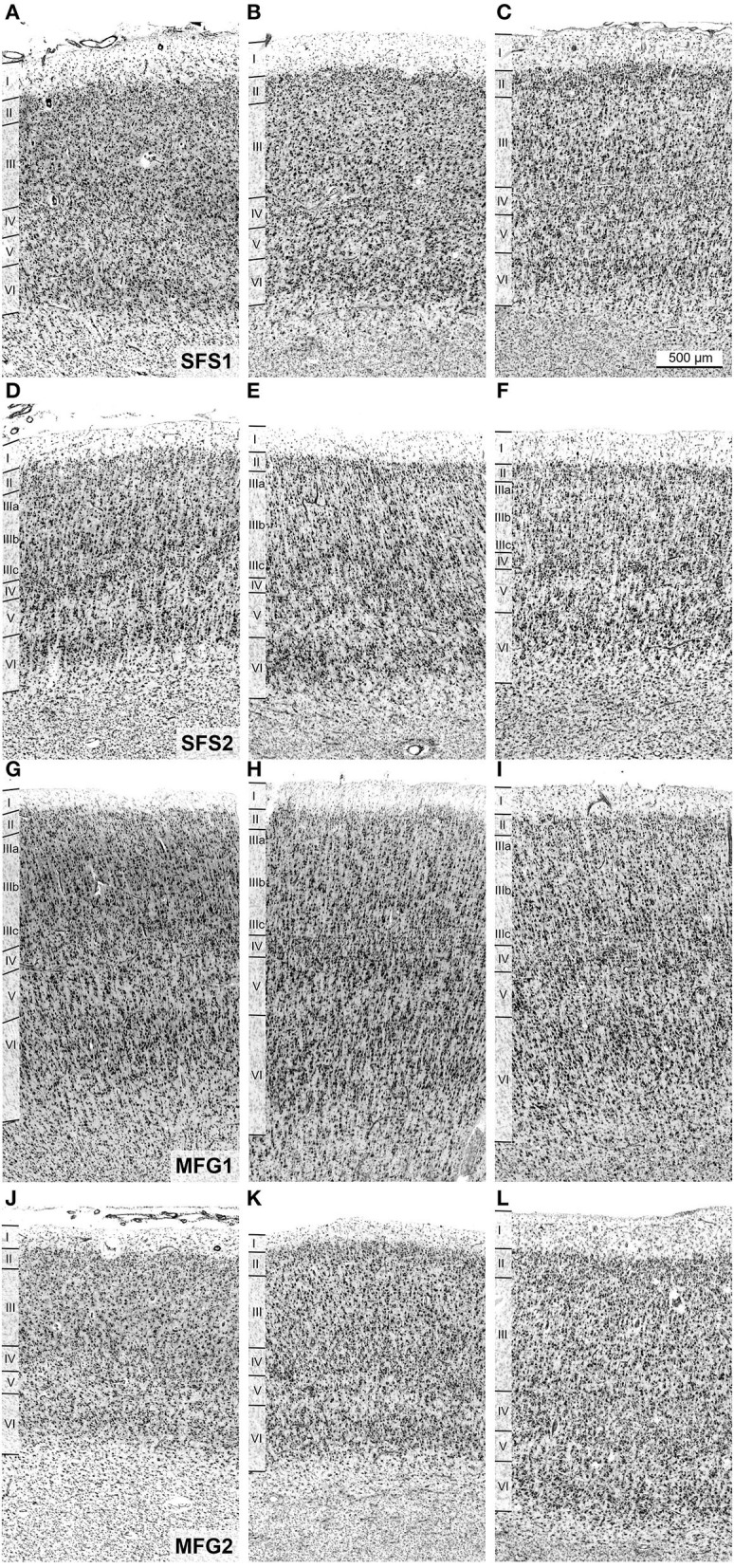
Cytoarchitecture and interindividual variability of anterior dorsolateral prefrontal cortex areas. Areas SFS1 **(A–C)**, SFS2 **(D–F)**, MFG1 **(G–I)**, and MFG2 **(J–L)** of three individual post-mortem brains are shown. Despite the intersubject variance between the individual brains, the decisive cytoarchitectonic characteristics can be recognized. For example, area SFS1 was characterized by a high cell density of layer II and distinct layers III and V with predominantly medium-sized pyramidal cells. Layer V was not subdividable into Va and Vb compared to adjacent area BA9 **(A–C)**. Area SFS2 mainly differed from SFS1 by a low cell density in upper layer IIIa and higher cell density in deeper layer IIIc with larger pyramidal cells in IIIc. Layer IV was poorly developed, and layer VI was prominent with a high cell density **(D–F)**. Area MFG1 was characterized by large pyramidal cells in deeper layer III and upper layer V, and layer IV was visible. There was no distinct border to the white matter **(G–I)**. In contrast, the cells in layers III and VI of area MFG2 were mostly equal in cell size and did not contain large pyramidal cells, resulting in a more homogeneous appearance than in other identified areas **(J–L)**. Scale bar of 500 μm in **(C)** refers to all **(A–L)**.

All identified areas showed six separable cortical layers, including layer IV, and, thus, represented a typical isocortex. However, individual areas differed from each other and neighboring areas by distinct cytoarchitectonic characteristics, like size, density, and arrangement of neurons within single cortical layers (Figures 3, 5).

**Figure 6:**
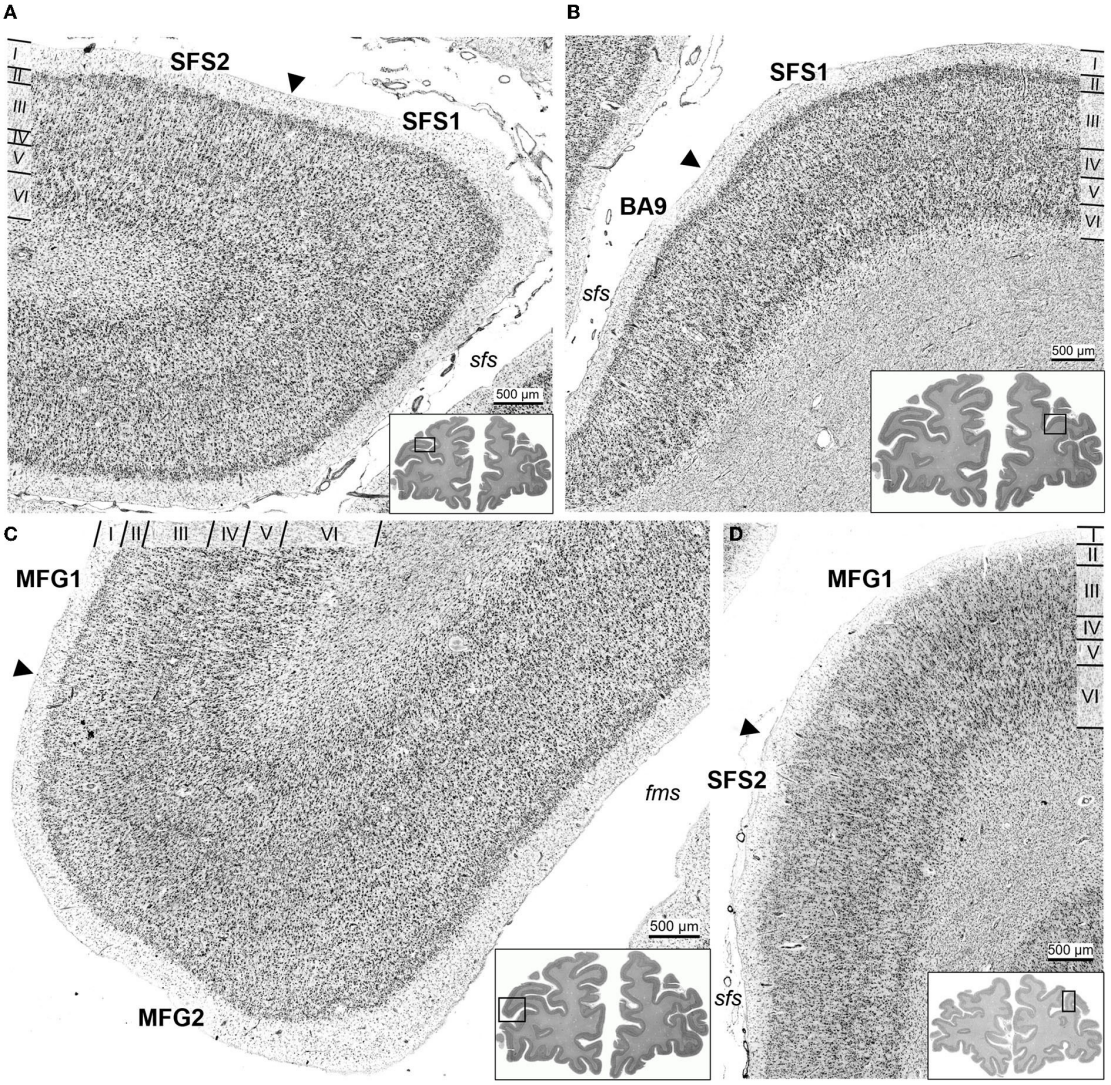
Cortical borders and cytoarchitecture of anterior dorsolateral prefrontal cortex areas. The characteristic feature of area SFS1 was a well-developed layer IV and a rather uniform appearance in general, due to similar cell density and size overall layers, compared to area SFS2 **(A)** and BA9 **(B)**. Area MFG1 had large pyramidal cells in deeper layer III and broad infragranular layers V and VI. In contrast, layers III and V of area MFG2 consisted of homogenous medium-sized cells and thin infragranular layers **(C)**. Area SFS2 had a loosely packed layer III, and a thin and blurred layer IV compared to SFS1 **(A)** and MFG1 **(D)**. Arrowheads indicate the respective cortical borders. Sulci labeled in italic (*sfs*, superior frontal sulcus; *fms*, frontomarginal sulcus).

In detail, SFS1 showed prominent, cell-dense, and well-developed layers II and IV, distinguishing it from neighboring BA9 and SFS2 (Figures 6A,B). Layer III consisted of small to medium-sized neurons with a slight cell-size gradient toward deeper layer III. Layer V with its medium-sized neurons, could not be subdivided into Va and Vb, as reflected by the flat curve in the GLI profile (Figure 3A). The border between layer VI and white matter was sharp compared to the neighboring areas BA9 and SFS2 (Figures 5A–C, 6A,B).

Area SFS2 had a thin layer II with no sharp border toward layer III. The main characteristic of area SFS2 was a very thin and blurry layer IV compared to SFS1 and MFG1. In the deeper layer IIIc, pyramid cells were larger and denser (represented by a local maximum in the GLI profile) than in upper layer IIIa (local minimum in the GLI profile), enabling the subdivision of layer III in IIIa, IIIb, and IIIc (Figure 3B). The medium- to large-sized cells in layer V were distributed less compactly in comparison to SFS1 and MFG1 and did not allow a clear subdivision into Va and Vb, as, for example, in BA9. Layer VI showed a high cell density, and the white matter border was more blurred than in SFS1, but sharper compared to MFG1 (Figures 5D–F, 6A,D).

Area MFG1 occupied the whole surface of the anterior *mfg* and was characterized by a larger cortical thickness in comparison to areas SFS1, SFS2, and MFG2. In terms of layering, MFG1 layer II was not as prominent and thick as in adjacent MFG2. As for SFS2, cell body sizes increased from upper to lower layer III, enabling a subdivision into IIIa, IIIb, and IIIc. However, cell density in MFG1 layer IIIa was lower in comparison to SFS1 and MFG2 (Figure 3C). Layer IV was in general visible, but the boundaries to layers III and V with their large neurons were blurry. However, layer IV had a higher cell density and was more pronounced than in adjacent cortical area SFS2 (Figure 6D). The infragranular layers V and VI were well-developed and occupied more than half the width of the entire gray matter. Layer V was rather uniform, with no clear subdivision. The broad and prominent layer VI consisted of densely packed large cells and a diffuse intersection to the white matter (Figures 5G–I).

**Figure 7:**
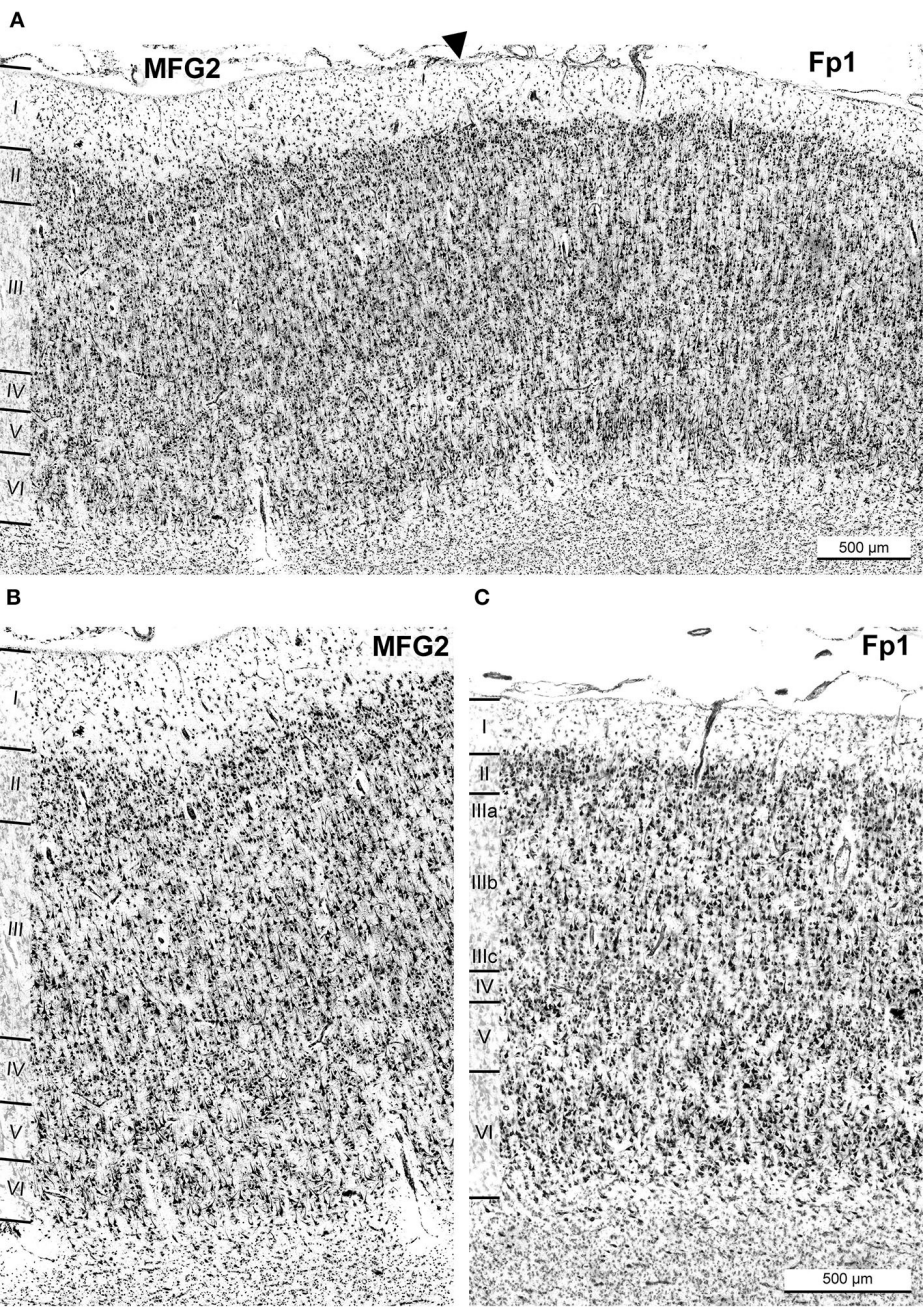
Cytoarchitectonic border of area MFG2 with neighboring frontal pole area Fp1. A black arrowhead indicates the border of MFG2 and Fp1 **(A)**. Comparison of cytoarchitectonic characteristics of area MFG2 **(B)** and Fp1 **(C)**. The most characteristic criteria for identifying MFG2 were a homogenous cell size across all layers with a broad layer II and a prominent cell-rich layer IV. The infragranular layers were thin and less developed and had a lower cell density than Fp1. The transition to white matter was sharp. Typical for Fp1 were large pyramidal cells in deeper than in upper layer III and a sharp border between layers I, II, III, and IV. Compared to MFG2, layer IV was not as well developed, and the infragranular layers V and VI were prominent. Scale bar 500 μm in **(C)** also refers to **(B)**.

Area MFG2 had a relatively homogenous cell density and cell size across all layers due to the absence of large pyramidal cells in layers III and V (Figure 3D). Thus, MFG2 could be clearly distinguished from adjacent areas of MFG1 and Fp1 (Figure 7). MFG2 layer II was dense and had a fluent transition into layer III. Layer IV was broad, cell dense, and well-developed. Large layer V pyramidal cells like in area MFG1 were almost absent, while cells in layer VI were densely packed. The transition to the white matter was sharp and, thus, different compared to the blurry white matter border of MFG1 (Figures 5J–L, 6C).

### Comparison with adjacent areas Fp1, BA9, and BA46

The cytoarchitecture of areas SFS1, SFS2, MFG1, and MFG2 differed from neighboring areas. Rostral to MFG2, the frontopolar area 1 (Fp1) was found (Bludau et al., 2014), which showed similarities and dissimilarities with the adjacent areas of the DLPFC (Figure 7A). Like MFG2, Fp1 had a dense layer II but with a sharper border to layer III. In stark contrast to MFG2, Fp1 showed a gradient of increasing cell body sizes from upper to lower layer III, with considerably larger pyramid cells deeper than in upper layer III, while cell size and distribution in MFG2 layer III were relatively homogenous (Figure 7B). Layer IV of Fp1 was not as broad and prominent as in MFG2, while the border between layer VI and the white matter appeared less sharp than in MFG2 (Figure 7C).

**Figure 8:**
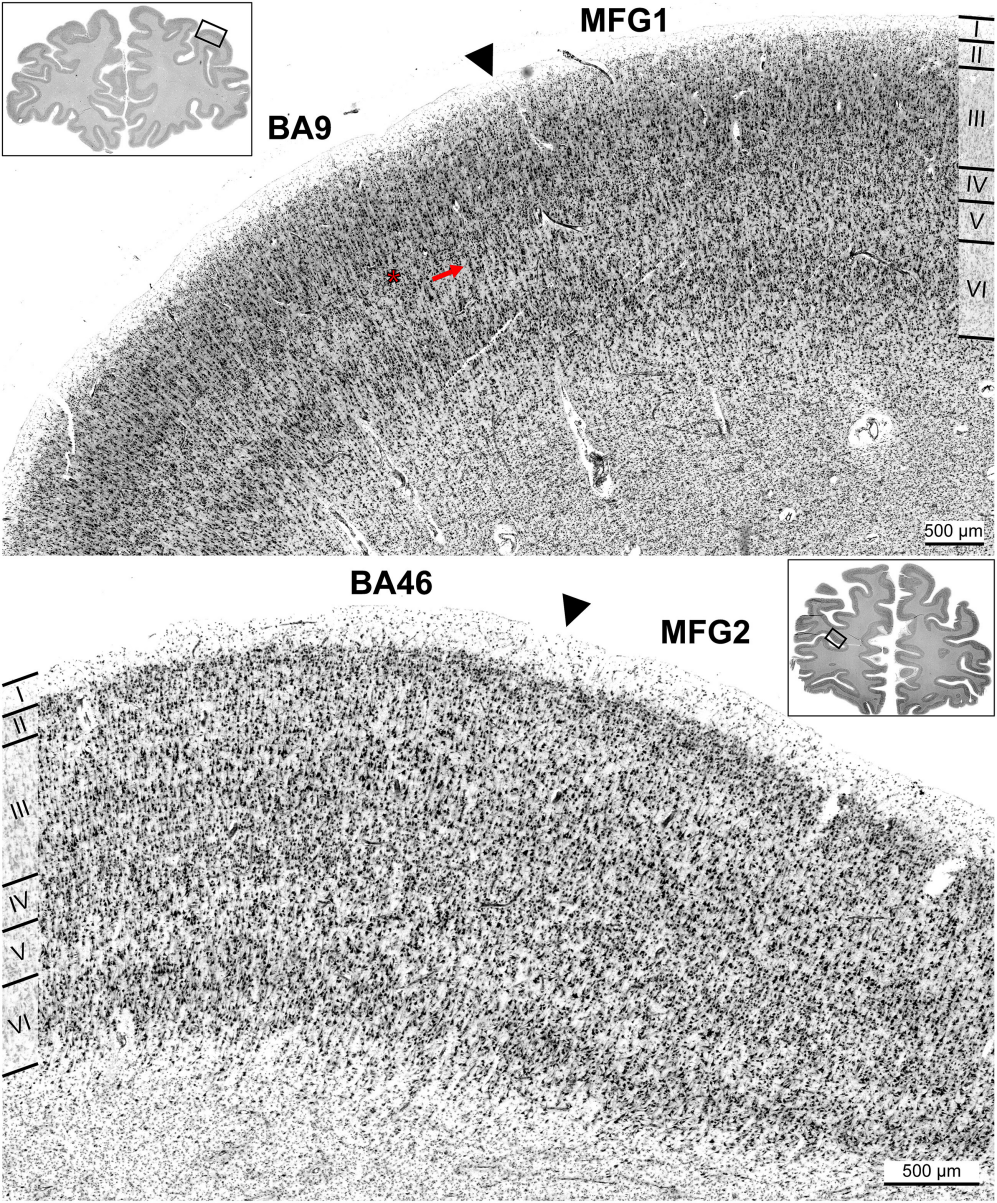
Cortical borders of anterior dorsolateral prefrontal cortex areas and adjacent areas. Area MFG1 shared a border with BA9 **(A)**. The main distinction of BA9 was a thin and blurred layer IV (red asterisk) and large pyramidal cells in layer Va (red arrow) compared to area MFG1 **(A)**. Area MFG2 also bordered ventrally to BA46, shown exemplarily in brain BC13 **(B)**. Typical for BA46 was the prominent layer IV. Layer III had a slight gradient in pyramidal cell size, and infragranular layers were more prominent than in area MFG2. Arrowheads indicate these borders.

Area BA9 (Rajkowska and Goldman-Rakic, 1995a) was located rostro-dorsally to area SFS1, mainly covering the descending part of the *sfs* and the surface of the superior frontal gyrus (Figure 6B). It was characterized by visible but thin layers II and IV, with layer IV displaying more fuzzy borders with adjacent layers III and V in comparison to SFS1. Like Fp1 and MFG1, BA9 layer III showed a gradient in cell body size with medium-sized to large neurons in deeper layer IIIc. The main cytoarchitectonic differences to area SFS1 were the generally lower cell density, which was especially low in layer IV, large pyramidal cells in layer Va, and a pale layer Vb. Further, the white matter border was not as sharp as in SFS1. In the caudal process of the *mfg*, a yet unmapped area appeared between areas SFS2 and MFG1 (Figure 8A). Cytoarchitectonically, this area resembled area BA9 because of very large pyramidal cells in V and a thin blurry layer in IV. This, in liaison with a blurry layer VI white matter border, allowed for a clear separation of this area from neighboring areas SFS2 and MFG1.

Ventrally to MFG2, a further yet unmapped area spanning the rising aspect of the sulcus was identified, which seems to correspond to parts of BA46 (Figure 8B). Compared to MFG2, this area had no homogenous appearance as the cell body size increased slightly across layer IIIa to layer IIIc. Further, the area showed a thin layer II with a diffuse transition to layer III. Layer IV consisted of densely packed granular cells and did not appear as prominent as in area MFG2 due to larger pyramidal cells in deeper layers III and V, blurring the boundaries between layers. Layer V, with loosely packed medium-sized pyramidal cells, and layer VI were well-developed and broad. The transition from layer VI to white matter was not as sharp as in area MFG2.

### Quantification of cytoarchitectonic differences and similarities of DLPFC areas

**Figure 9:**
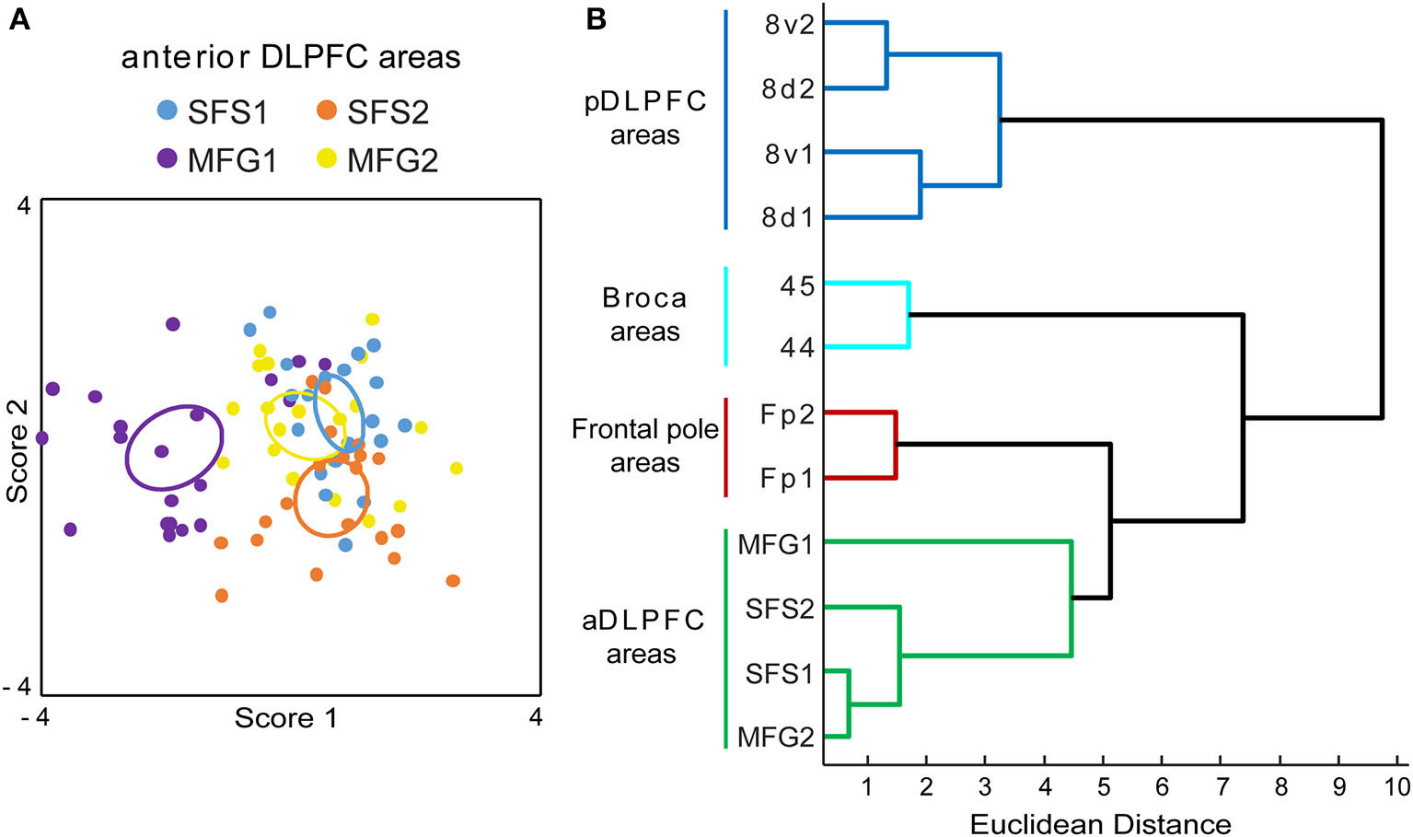
Discriminant and cluster analysis of Gray Level Index (GLI) profiles. The GLI profiles of all anterior dorsolateral prefrontal cortex (aDLPFC) areas were compared in a discriminant analysis **(A)**. Each area is represented by a set of 20 dots (2 hemispheres of 10 brains) and an ellipsoid, indicating the centroid for each area. The variance in the localization of the dots reflects the cytoarchitectonic intersubject variability. Area MFG1 (purple) and area SFS2 (orange) can be separated from the areas SFS1 (blue) and MFG2 (yellow). The dendrogram of the hierarchical cluster analysis **(B)** separates the aDLPFC areas from the frontal pole areas Fp1 and Fp2 (Bludau et al., 2014), Broca areas 44 and 45 (Amunts et al., 2004), and areas of the posterior DLPFC (pDLPFC) 8v1, 8v2, 8d1, and 8d2 (Amunts et al., 2021). aDLPFC areas form a distinguishable separate cluster, representing structural differences compared to their adjacent areas in the prefrontal cortex. A high Euclidean distance on the x-axis indicates structural dissimilarity.

Areas of the anterior DLPFC SFS1, SFS2, MFG1, and MFG2 were separated in a discriminant analysis using GLI profiles (Figure 9A). The analysis revealed that even though GLI profiles showed some interindividual variance, all identified areas form discrete clusters, with only an intersection of cluster centroids for SFS1 and MFG2. Area MFG1 formed a cluster that is separated from the other areas SFS1, SFS2, and MFG2. The same was true for area SFS2. Area SFS2 was cytoarchitectonically more similar to areas SFS1 and MFG 2 than neighboring area MFG1. Area SFS1 cytoarchitectonically resembled area MFG2, indicated by the slight intersection. However, we were able to detect and verify the borders between these areas consistently along the area's progress.

In the hierarchical cluster analysis, the anterior DLPFC areas SFS1, SFS2, MFG1, and MFG2 were compared with neighboring areas of the prefrontal cortex, i.e., frontal pole areas Fp1 and Fp2 (Bludau et al., 2014), areas 44 and 45 of Broca's region (Amunts et al., 2004), and the posterior DLPFC areas 8v1, 8v2, 8d1, and 8d2 (Amunts et al., 2021). The newly defined areas in the anterior DLPFC showed smaller distances to the frontal pole areas Fp1 and Fp2 than to areas of Broca's region and the posterior DLPFC areas based on the Euclidean distance measure (Figure 9B).

To examine whether and how the observed volume differences between male and female brains were related to the underlying cytoarchitecture, we analyzed the volume fraction of cell bodies reflected by GLI values for each region with a mixed model ANOVA with repeated-measures design (within factors, area and hemisphere; between factor, sex). However, the analyzed GLI between the two hemispheres and sexes did not reach statistical significance (*p* > 0.55).

### Probability maps and maximum probability map

**Figure 10:**
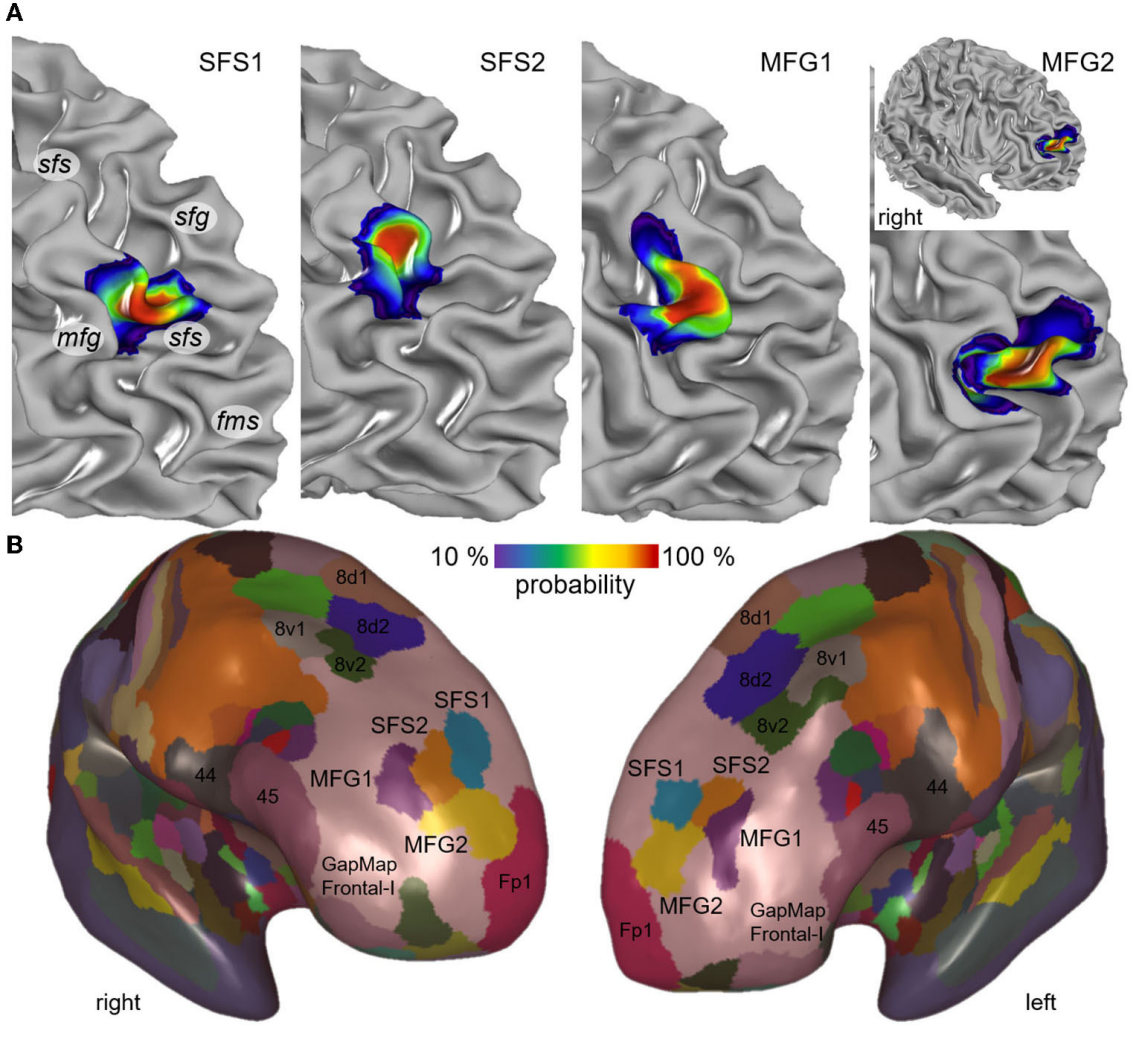
Maximum probability map and probability maps of the newly identified areas. The individual probability maps of the new areas SFS1, SFS2, MFG1, and MFG2 are illustrated on the right prefrontal hemisphere of the stereotaxic MNI Colin27 template brain **(A)**. Maps are depicted in smooth white matter mode to demonstrate the area localization on sulci and gyri (*sfg*, superior frontal gyrus; *sfs*, superior frontal sulcus; *mfg*, middle frontal gyrus; *fms*, frontomarginal sulcus). The probability maps indicate color-coded interindividual variability. Values from 10 to 100% (blue to red) describe the degrees of overlap, e.g., red regions correspond to at least 82% probability. The non-overlapping surface representation of MNI Colin27 illustrates the position of SFS1 (blue), SFS2 (orange), MFG1 (purple), and MFG2 (yellow) in conjunction with the neighboring frontal pole area Fp1 (magenta), posterior DLPFC areas (8d1, 8d2, 8v1, and 8v2) and areas of Broca's region (44 and 45) on an inflated brain surface **(B)**. The newly identified areas were located in the GapMap Frontal-I (rose) of yet unmapped prefrontal cortex areas. Probability map and maximum probability map are publicly available at: https://jubrain.humanbrainproject.eu.

The high interindividual variability of the anterior part of the DLPFC of the individual brains and the location of new areas SFS1, SFS2, MFG1, and MFG2 are shown in Figure 4. Cytoarchitectonic probability maps in the two anatomical reference spaces MNI Colin27 (Figure 10A) and ICBM152casym were calculated to quantify the interindividual variability in the stereotaxic localization and extent of the four anterior DLPFC areas. Centers of gravity of anterior DLPFC areas are provided in Table 3 for MNI Colin27 and ICBM152casym.

**Table 3:** Center of gravity coordinates in anatomical MNI Colin27 and MNI ICBM 152 space of anterior DLPFC areas separated by hemisphere.

**Area**	**Hemisphere**	**MNI Colin27 space**			**ICBM152casym space**		
		**x**	**y**	**z**	**x**	**y**	**z**
SFS1	Left	−25	48	22	−27	50	21
	Right	26	50	20	25	52	21
SFS2	Left	−29	46	27	−31	47	25
	Right	30	48	22	28	50	23
MFG1	Left	−36	50	23	−38	50	20
	Right	39	48	22	37	50	21
MFG2	Left	−25	52	18	−26	53	17
	Right	31	52	11	30	55	11

The visualization of the probability maps showed that the descending part and fundus of the rostral *sfs* were covered by area SFS1. In contrast, area SFS2 was located on the ascending part of the *sfs*, reaching partly to the surface of *mfg* but with decreasing probability. Area MFG1 was predominately located on the surface of the *mfg*, reflected by the large overlap in all ten brains. The ascending and descending sulci parts adjacent to the *mfg* have less overlap than the *mfg* surface and, thus, greater interindividual variability. Ventrally to area MFG1, area MFG2 was located in a caudal extension of the *fms* or the anterior *mfs*, if existing. This variance in location is reflected by higher interindividual variability in both hemispheres compared to the other areas (Figure 10A).

A non-overlapping surface representation of all four anterior DLPFC areas is provided by the MPM, which shows the topography of the four new areas and the cytoarchitectonically delineated adjacent areas Fp1, the posterior DLPFC areas 8d1, 8d2, 8v1, and 8v2 and areas 44 and 45 of the ventral prefrontal cortex on the inflated brain surface of MNI Colin27 (Figure 10B). Area MFG2 is bordered rostrally by the frontal pole area Fp1. The newly identified areas are located in an extensive unmapped region in the frontal brain region described as “GapMap Frontal-I” (Amunts et al., 2020).

The new maps are publicly available, free to share and adapt under the creative commons license agreement, and open for download at https://ebrains.eu/.

### Volumes of areas SFS1, SFS2, MFG1, and MFG2 in the anterior DLPFC

**Figure 11:**
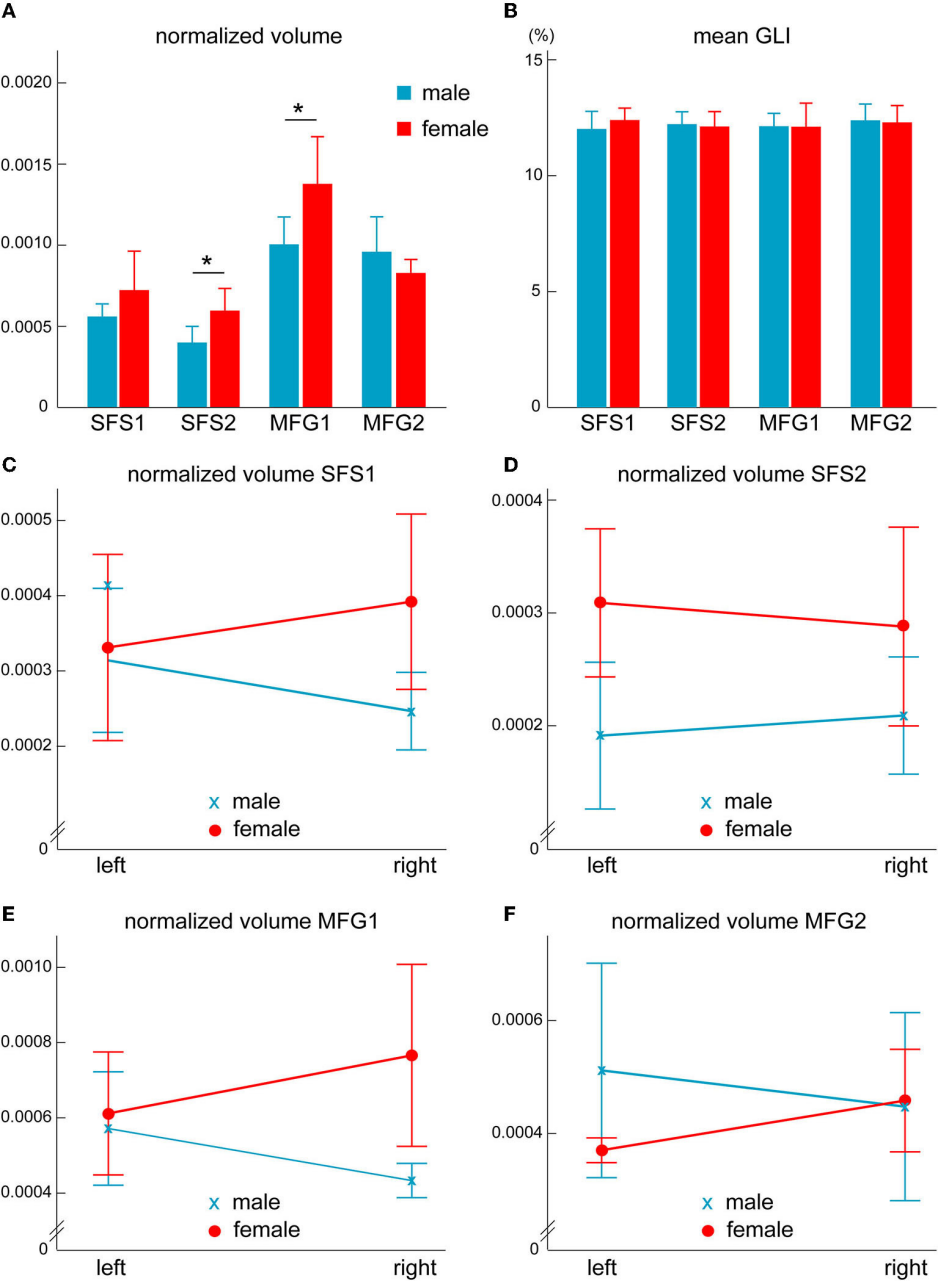
Sex differences in anterior dorsolateral prefrontal cortex areas. Normalized volumes of areas SFS2 and MFG1 differ significantly between sexes [*p < 0.05, **(A)**]. However, no sex differences were found in the volume fraction of cell bodies **(B)**. Analysis divided by hemispheres **(C–F)** revealed significantly higher area volumes (*p* < 0.05) in female than in male brains in the right hemisphere of area SFS1 **(C)** and MFG1 **(E)** and in the left hemisphere of area SFS2 **(D)**. Normalized area volumes and GLI are presented as Mean ± SD.

Differences in shrinkage-corrected volumes of the four areas were analyzed concerning interhemispheric and sex differences (Figure 11). Area MFG1 showed the largest volume (1,392 ± 278 mm3), followed by MFG2 (1,069 ± 281 mm3), SFS1 (754 ± 201 mm3), and SFS2 (578 ± 142 mm3). The combined cortical volume of anterior DLPFC areas in the right hemisphere was 1,889 ± 348 mm3 and 1,903 ± 419 mm3 in the left (*p* = 0.938). Male brains had a total volume of 3,748 ± 695 mm3, with 1,714 ± 378 mm3 in the right and 2,034 ± 496 mm3 in the left hemisphere (*p* = 0.283). Female brains had a total volume of 3,835 ± 378 mm3, with 2,064 ± 231 mm3 in the right and 1,771 ± 324 mm3 in the left hemisphere (*p* = 0.140).

The shrinkage-corrected area volumes were normalized to the corresponding total brain volume and then compared using an ANOVA to identify putative sex differences. Both groups (males and females) were normally distributed, sphericity, and homogeneity of the error variance were given, as assessed by the Shapiro–Wilk test (*p* > 0.05), the Mauchly's test (*p* > 0.05), and the Levene's test (*p* > 0.05), respectively. The ANOVA (within factor, area; between factor, sex) revealed that anterior DLPFC areas showed area-specific sex differences [*F*_(3, 24)_ = 3.946, *p* < 0.021]. Subsequent univariate *F*-tests showed that areas SFS2 (*p* < 0.035) and MFG1 (*p* < 0.046) were significantly larger in females than in male brains, while differences in the other areas did not reach significance (SFS1: *p* = 0.211, MFG2: *p* = 0.260; Figure 11A).

To study putative lateralization effects, we further analyzed the normalized area volumes with a mixed model ANOVA with a repeated-measures design (within factors, area and hemisphere; between factor, sex). Volumes were normally distributed (Shapiro-Wilk test, *p* > 0.05), and sphericity and homogeneity of the error variances were given (Mauchly's test and Levene's, both *p* > 0.05). The ANOVA revealed a significant difference in area-by-sex-interaction [*F*_(3, 24)_ = 3.946, *p* < 0.021, partial η^2^ = 0.330]. Subsequent univariate *F*-tests showed significant volume differences between areas of male and female brains in the right area SFS1 (*p* < 0.047; Figure 11C), left SFS2 (*p* < 0.022; Figure 11D), and right MFG1 (*p* < 0.036; Figure 11E), with larger volumes in female than in male brains (Figure 11 and Table 4).

**Table 4:** Shrinkage corrected volumes of areas **(mm**3**)**.

**Brain No**.	**Sex**	**Left**				**Right**			
		**SFS1**	**SFS2**	**MFG1**	**MFG2**	**SFS1**	**SFS2**	**MFG1**	**MFG2**
BC04	Male	285	209	518	239	235	254	451	469
BC11	Male	358	269	956	598	341	362	672	978
BC13	Male	550	354	852	791	232	263	465	531
BC20	Male	321	179	521	885	438	277	591	431
BC21	Male	492	196	798	799	346	174	603	454
Mean		400	241	731	662	318	266	557	573
SD		116	72	202	259	87	67	95	229
BC05	Female	601	403	787	404	655	399	803	368
BC08	Female	279	269	713	463	355	216	667	496
BC09	Female	327	357	894	404	374	219	910	491
BC10	Female	253	254	477	387	332	329	1,156	517
BC14	Female	347	399	476	362	422	396	595	617
Mean		362	336	669	404	428	312	826	498
SD		139	71	187	37	131	91	221	89

## Discussion

This study identified four new cytoarchitectonically distinct areas (SFS1, SFS2, MFG1, and MFG2) within the human anterior DLPFC, applying an observer-independent histological mapping approach. This method allowed us to map the new areas in a reproducible way and quantify cytoarchitectonic differences and similarities based on statistical tests. A new nomenclature was introduced since the present data revealed a more fine-grained parcellation of the anterior DLPFC as previously reported, and to avoid assumptions regarding correspondences with results of earlier classifications. The new areas varied between brains concerning their precise relationship to sulci and gyri, as well as in localization and extent in 3D space. This variability was captured by 3D cytoarchitectonic probability maps in both ICBM152casym and MNI Colin27 space. The maps enable the direct comparison with results from functional imaging studies to address the functional parcellation of this region.

### Structural-functional properties of the human prefrontal cortex

A major challenge of any investigation of this region is the sulcal pattern, which is highly variable among brains. For example, the presence of the *mfs* varied between brains and hemispheres (Ono et al., 1990). When no *mfs* was present, for example, in the right hemisphere of BC05 (Figure 4), area MFG2 was located in the caudal extension of the *fms*. This anatomical variance may contribute to some ambiguity regarding sulcal labeling and definition by different authors, especially concerning the *mfs*. Recent segmentations propose subdivision of the *mfs* in posterior (*pmfs, posterior middle frontal*) and anterior (*imfs, intermediate frontal*) components (Petrides, 2019; Miller et al., 2021b), whereas the latter ones consist of a horizontal (*imfs-h*) and vertical (*imfs-v*) part, synonymous with the *fms*, and consistent with the classical definition of *mfs* (Eberstaller, 1890). Moreover, Miller et al. (2021a,b) propose that these tertiary sulci may serve as landmarks in the prefrontal cortex, linking microstructure and functional features. This study supports the notion that cytoarchitectonic boundaries cannot be delineated based on anatomical landmarks exclusively (Amunts et al., 2004) due to high macroanatomical variance in the DLPFC and varying relationships of cortical areas concerning the sulcal patterns (see Figure 4). Previous cytoarchitectonic maps divided the whole DLPFC in two (Brodmann, 1909; Sarkissov et al., 1955) or, at most, four areas (von Economo and Koskinas, 1925; Rajkowska and Goldman-Rakic, 1995a; Petrides and Pandya, 1999).

Using the newly identified areas SFS1, SFS2, MFG1, and MFG2 as part of the Julich-Brain allows for the comparison of probability maps with results from *in vivo* neuroimaging studies and connectivity analyses to facilitate further exploration of the microstructural correlates of a variety of brain functions.

Interestingly, Friedman and Robbins (2022) reviewed the models and concepts of cognitive control and suggested a diversity, as well as the unity of prefrontal cortex functions, which may reflect the still controversially discussed functional segregation of the prefrontal lobe (Stuss, 2011; Goulas et al., 2012; Cieslik et al., 2013; Reid et al., 2016). Stuss (2011) subdivided the prefrontal lobe into the following functional categories: “dorsomedial for energization, left dorsolateral for task setting, and right dorsolateral for monitoring…ventral-medial/orbital for emotional and behavioral self-regulation, and frontopolar for integrative -even meta-cognitive- functions.” This is partly in line with prior models that functionally subdivided the prefrontal cortex along a dorsal-ventral axis (Petrides, 2005), like, for example, the “What vs. How” theory of O'Reilly (2010). More recent investigations seem to confirm an axis-orientated prefrontal cortex organization (Cieslik et al., 2013; Glasser et al., 2016a; Nee and D'Esposito, 2016). Glasser et al. (2016a) for example, also subdivided the DLPFC into anterior and posterior subdivisions in a multimodal parcellation study based on connectivity, microstructure, and function.

A further study comparing whole-brain co-activation patterns across neuroimaging studies also subdivided the right DLPFC into anterior-ventral and posterior-dorsal subregions (Cieslik et al., 2013). They found that while both subregions are involved in distinct neural networks, only the anterior one is associated with attention and action inhibition. Activation in the anterior network was also found in tasks requiring conflict resolution like the Stroop task and Go/No-Go task (Cieslik et al., 2013). Cieslik et al. (2013) could even further subdivide the anterior cluster (center of gravity MNI coordinate: x = 30, y = 43, z = 23) into a rostral and a caudal part at a lower hierarchical linkage level. When comparing MNI coordinates of these subclusters with our newly identified areas, SFS1 and SFS2 overlapped to a large extent with the rostral and caudal anterior subclusters of Cieslik et al. (2013), revealing the assignment of our microstructural maps with functional parcellations.

The new areas may also shed new light on assignments in the most anterior part of the DLPFC that were attributed to frontal pole area 10 (Wager et al., 2005; Chevrier et al., 2015; Shafritz et al., 2015; Crane et al., 2016). For example, Sallet et al. (2013) described an anterior lateral cluster (center of gravity MNI coordinate: x = 31, y = 48, z = 11) based on an MRI parcellation studying structural connectivity that was formerly assigned to the frontal pole area. However, having a much larger ventral extent, the cluster defined by Sallet et al. fits a large degree of the newly identified anterior MFG2, but not to the frontal pole region. Such studies of structure-function relationship may benefit in the future from cytoarchitectonic probability maps of the DLPFC and provide more precise microstructural correlates of functional activation patterns and maps.

The new maps are accessible through the multilevel Human Brain Project (HBP) atlas and are available at the EBRAINS digital research infrastructure under https://ebrains.eu/service/human-brain-atlas/. In this environment, the maps are linked to complementary brain data like the atlas of fiber bundles (Guevara et al., 2017) and dictionaries of functional modes (DiFuMo atlases; Dadi et al., 2020). Dadi et al. summarized and extracted millions of fMRI studies signals in a finely resolved atlas of functional modes ranging from 64 to 1,024 functional networks. Comparing our data with the coordinates of the classified domains of the highest resolution DiFuMo atlas with 1,024 components reveals interesting correspondences. Regarding the left hemisphere, area SFS1 seems to correspond to component 994 (x = −23, y = 54, z = 19), SFS2 to component 834 (x = −30, y = 49, z = 20), MFG1 to component 428 (x = −39, y = 46, z = 23), and MFG2 to component 844 (x = −29, y = 53, z = 8; Dadi et al., 2020). These results support the hypothesis that the identified areas are closely linked to specific functional networks and, thus, show the potential applicability of our new microanatomical maps.

### Interpretation of anterior DLPFC areas in the context of previous cytoarchitectonic maps

As described above, previous cytoarchitectonic maps do not reflect the heterogeneity of the DLPFC that can be assumed from functional parcellations. Brodmann (1909) and Sarkissov et al. (1955) subdivided the human DLPFC into two distinct areas, areas 9 and 46, but with slightly different extent and neighborhood relationships compared to, von Economo and Koskinas (1925) and Sarkissov et al. (1955).

Rajkowska and Goldman-Rakic (1995a) later supplemented their microscopic observations of areas 9 and 46 with morphometric data using quantitative area criteria, like cortical and laminar thickness and neuronal soma size. Thereby, they provided an unbiased cytoarchitectonic analysis of the DLPFC with objective criteria, and we will, thus, focus the comparison of our data to these parcellations and the parcellations of Petrides and Pandya (1999). Area 9 of Rajkowska and Goldman-Rakic is mainly located on the superior frontal gyrus and is characterized by a clear sublimination of layers III and V, with large pyramidal cells in IIIc and Va. The narrow layer VI is loosely packed, and the transition from layer VI to white matter is indistinct. Their area 46 is situated on the *mfg*, mainly extending to the depth of the *mfs*. Its main characteristic is a pronounced layer IV and a homogenous size and compact arrangement of cells, especially in layer III. Layer VI is thick and can be divided into two distinct sublayers.

When comparing the cytoarchitectonic descriptions of Rajkowska and Goldman-Rakic to the new anterior DLPFC areas, a high concordance to our areas SFS1 and MFG2 is evident. Both have a pronounced, densely packed layer IV, and a relatively uniform appearance due to the homogenous cell sizes and compact arrangements, particularly in area MFG2. This is also in agreement with the cytoarchitectonic characteristics listed for area 46 by Petrides and Pandya (1999). However, in contrast to the map of Petrides and Pandya, this region is subdivided into the areas SFS1 and MFG2 in our new maps, based on the finding that layer II of area MFG2 is not as densely packed, and layer IV is slightly broader as in SFS1. These differences have been verified by the observer-independent mapping procedure. Furthermore, the pyramidal cells in layer V of area MFG2 are less dense and smaller compared to SFS1. In contrast to the delineation of area 46 of Rajkowska and Goldman-Rakic, we did not find a consistent subdivision of layer VI, and our area SFS1 is localized in the depth of *sfs* and not on the surface of *mfg* or within the *mfs*. Most likely, “individual variation […] observed in the structure of area 46” as described by Rajkowska and Goldman-Rakic (1995a, p. 310), rather indicate the existence of different areas.

Area MFG1, mainly occupying the surface of the *mfg*, has a well-developed layer IV, and the infragranular layers V and VI are broad and can be further subdivided. In addition, pyramidal cells of layer III differ considerably in cell size, with smaller neurons in IIIa and larger pyramidal cells in deeper layer IIIc. This fits well with the description of the dorsal part of area 9/46 as defined by Petrides and Pandya (1999) and Petrides et al. (2012). They interpreted area 9/46 because of its localization on the *mfg* as corresponding to area 9 (e.g., by Brodmann, 1909; Sarkissov et al., 1955), while it showed more cytoarchitectonic similarity to area 46. Area 9/46d, lying on the *mfg*, has large pyramidal neurons in deeper layer III, but not as many as in the ventral part 9/46d. Layer IV is well-developed, layer V contains medium-sized pyramidal cells, and layer VIa can be separated by the sparse layer VIb (Petrides and Pandya, 1999). These criteria mainly agree with the present cytoarchitectonic description of area MFG1, whereas the areas 9 and 46 descriptions of Rajkowska and Goldman-Rakic (1995a) do not seem to fit.

A clear assignment of our area SFS2 to areas from previous studies seems to be challenging. The main features of area SFS2 are a gradient in cell body size across layer III, as well as a visible but blurry layer I, with intermingling large pyramidal cells of deep layer III and layer V. Thus, area SFS2 displays some cytoarchitectonic properties of area 9 of Rajkowska and Goldman-Rakic (1995a). However, we did not observe a compact layer II, nor very large pyramidal cells in upper layer V as described by Rajkowska and Goldman-Rakic (1995a), as well as Petrides and Pandya (1999). As area 9 has a large extension described by Rajkowska and Goldman-Rakic and “[s]ome variations in the basic cytoarchitectural pattern of area 9 were often evident […] in the rostrocaudal axis” (Rajkowska and Goldman-Rakic, 1995a, p. 309), it is likely that there is no “single” area 9 but several independent areas (like area SFS2) that lie in the region of the initially defined area 9 by Rajkowska and Goldman-Rakic.

Based on this study, we conclude that a comparable detailed structural parcellation of the DLPFC exists, as functional studies have already demonstrated. However, their precise relationship is a topic of future research. This conclusion needs to be also further evaluated when cytoarchitectonic mapping of the remaining parts of the DLPFC is progressing. Recently, the yet uncharted regions in the more dorsal and more ventrally located parts of the DLPFC are summarized in “GapMap Frontal-I” (Amunts et al., 2020). However, the delineation of neighboring areas in the current work already indicates that the relatively simple subdivision of the human DLPFC into the two areas 9 and 46 seems to be insufficient to reflect the microstructural parcellation adequately and likely needs to be supplemented.

### Sex differences in anterior DLPFC areas

Most of the analyzed parameters concerning cytoarchitecture (e.g., microstructural characteristics and mean GLI profiles) and area localization of the DLPFC areas did not differ significantly between male and female brains, suggesting a rather identical cytoarchitecture of areas and related function. Interestingly, a statistically significant higher absolute volume in areas SFS2 and MFG1 was found in females as compared to male brains, although the latter showed a larger total brain volume. Additionally, the area volumes showed area-specific sex differences with higher volumes in females as compared to male brains. To our knowledge, such differences have been shown for the first time. Previously published cytoarchitectonic studies of the prefrontal cortex, i.e., areas in the lateral orbitofrontal cortex (Wojtasik et al., 2020) and frontal pole areas Fp1 and Fp2 (Bludau et al., 2014), did not indicate differences between male and female brains. However, sex differences in anatomical measures of asymmetry (e.g., volume or surface) have been described for some other brain regions, including the Broca's region (Amunts et al., 1999) and visual areas (Amunts et al., 2007).

In contrast, evidence has been provided for sex differences on a macroscopical scale, like larger gray matter volume, white matter volume, and total brain volume, mainly in men, but also in brain regions where women showed increased values (i.e., frontoparietal cortex; Ide et al., 1996; Giedd et al., 2012; Ruigrok et al., 2014; Ritchie et al., 2018; Lotze et al., 2019). For example, a meta-analysis of Ruigrok et al. showed that gray matter volume in the right *mfg* and left frontal pole are larger in female brains. Furthermore, there was an asymmetry within these larger volumes in females, mainly in the right hemispheres (Ruigrok et al., 2014). The present data are in accordance with this observation that volumes in our anterior DLPFC areas were larger in females, especially with a trend to the right hemisphere. Hemispheric asymmetry occurs in both sexes, with the cortical volume of the right hemisphere being more extensive than that of the left (Zilles, 1972). Structural asymmetries favoring the left hemisphere are described for several brain regions, like the inferior frontal gyrus (i.e., Broca's region; Amunts et al., 1999, 2003), central sulcus (Cykowski et al., 2008), the planum temporale (Galaburda et al., 1978), cingulate cortex (Wang et al., 2007), and anterior cingulate cortex (Huster et al., 2007). Fewer studies described right-over-left asymmetry in the human brain (Murphy et al., 1987; Amunts et al., 2007). Regarding the frontal lobe, the right hemisphere seems to be wider than the left one (Toga et al., 2009). Thus, the wider right prefrontal lobe might contribute to our observed trend of right-over-left asymmetry in DLPFC areas in females.

There is evidence that males and females make use of different strategies to solve various tasks (Boghi et al., 2006; Li et al., 2006; Christakou et al., 2009; Rubia et al., 2013; Yuan et al., 2019). For example, several studies revealed a better performance during construction tasks and activities involving spatial cognition and spatial learning in males (Geary et al., 2000; Saucier et al., 2002; Voyer et al., 2007). On the other hand, females showed increased frontal activations during attention, cognitive switching, and verbal and memory tasks in comparison to males (Fenson et al., 1994; Kramer et al., 1997; Bell et al., 2006; Christakou et al., 2009; Rubia et al., 2010). Furthermore, they showed better performance in social skills, i.e., empathy and facial expression sensitivity, probably due to a better-developed theory of mind (Dunn et al., 1991; Baron-Cohen, 2002). Presumably, the larger volumes in the anterior DLPFC areas in female brains found in this cytoarchitectonic study reflect parts of these sex differences and may be related to behavioral differences between sexes, but more precise effects need to be clarified by future research. Furthermore, our results do not exclude the possibility that sex-related differences exist at a finer microstructural level, for example, in individual layers, or terms of the number of glial cells, but they demonstrate that volumetric differences do not stem from gross differences in volume fraction of cell bodies across all layers.

As a closing remark, the limitation of this study by the rather low sample size compared to MR studies has to be mentioned. Our systematic mapping study offers high spatial resolution but is, therefore, labor-intensive, and highly time-consuming, limiting the analyzed sample size. This may result that certain area, interhemispheric, and/or sex differences did not reach significance because of substantial interindividual variability.

## Conclusion

This study revealed cytoarchitectonically four new areas (SFS1, SFS2, MFG1, and MFG2) in the anterior region of DLPFC with a quantitative image analysis approach. The 3D reconstructions of newly delineated areas illustrate the high interindividual variability and the complex and variable sulcal pattern of the prefrontal cortex. It was found that the human DLPFC is cytoarchitectonically finer segregated than was previously assumed. Therefore, the simplified concept of the “one DLPFC” must be extended. We assume that the new areas are specifically integrated into functional networks, as comparisons of our data with the DiFuMo atlas (Dadi et al., 2020) revealed striking agreements. The generated probability maps account for interindividual variability and are part of the HBP atlas. They provide a profound cytoarchitectonic basis that will be complemented by future mapping studies like analyses of receptor or myelin architecture. The specific cytoarchitectonic characteristics of the new delineated areas may help further investigate laminar-specific differences as observed in layer III, especially in the context of schizophrenia (Arnsten et al., 2022). Furthermore, the new maps are linked to toolboxes (i.e., “siibra toolsuite” Dickscheid, 2021) that enable, for example, comparison with neuroimaging data. Thus, our new fine-resolved microstructural maps provide an improved anatomical base for future interpretation of functional data and help elucidate the still controversially discussed organizational principle of the human prefrontal cortex.

## Data availability statement

The original contributions presented in the study are included in the article, further inquiries can be directed to the corresponding author.

## Ethics statement

The used post-mortem tissue in this study was obtained through the body donor program of the medical faculty of the Heinrich-Heine-University Düsseldorf and approved by the Ethics Committee of the same institution (# 4863).

## Author contributions

AB cytoarchitectonically mapped and analyzed anterior DLPFC areas SFS1, SFS2, MFG1, and MFG2, performed the final calculation and statistical analysis, and wrote the manuscript under the supervision of KA. The study was designed by KA. SB calculated the hierarchical cluster analysis. HM calculated the maps in reference space and estimated volumetric analysis. All authors contributed to the manuscript and approved its publication.

## Funding

This project has received funding from the European Union's Horizon 2020 Framework Programme for Research and Innovation under the Specific Grant Agreement No. 945539 (Human Brain Project SGA3).

## Conflict of interest

The authors declare that the research was conducted in the absence of any commercial or financial relationships that could be construed as a potential conflict of interest.

## Publisher's note

All claims expressed in this article are solely those of the authors and do not necessarily represent those of their affiliated organizations, or those of the publisher, the editors and the reviewers. Any product that may be evaluated in this article, or claim that may be made by its manufacturer, is not guaranteed or endorsed by the publisher.
